# Editorial: New ideas in Performance Science

**DOI:** 10.3389/fpsyg.2024.1504522

**Published:** 2024-10-24

**Authors:** Vassilis Sevdalis, Niels Chr. Hansen, Valentin Bégel

**Affiliations:** ^1^Department of Food and Nutrition and Sport Science, University of Gothenburg, Gothenburg, Sweden; ^2^Department of Music, Art and Culture Studies, Centre of Excellence in Music, Mind, Body and Brain, University of Jyväskylä, Jyväskylä, Finland; ^3^Interacting Minds Centre, School of Culture and Society, Aarhus University, Aarhus, Denmark; ^4^Royal Academy of Music Aarhus, Aarhus, Denmark; ^5^Institut des Sciences du Sport Santé de Paris (I3SP), URP 3625, Université Paris Cité, Paris, France

**Keywords:** performance, skills, individual differences, teaching, learning, context, interaction, health and well-being

## 1 Introduction

This Research Topic was launched to serve as a compendium of forward-looking contributions from basic and applied research, outlining recent developments, novel ideas, and directions within the fast-growing field of Performance Science. This interdisciplinary research field encompasses topics and approaches from diverse domains, and provides insights into fundamental skills, psychological and physiological mechanisms, and outcomes of performance activities and experiences (e.g., Cotterill, 2017; Mugford and Cremades, 2018; Murphy, 2012). Scientific advances in Performance Science foster performance by enabling the development of innovative interventions tailored toward aspects of education, training, health, and wellbeing across domains of human performance (e.g., Bégel et al., 2024; Sevdalis and Wöllner, 2016; Williamon, 2004).

The 17 contributions comprise: Thirteen Original Research articles, two Opinion articles, one Hypothesis and Theory article, and one Review article. Collectively, these articles showcase the breadth and depth of Performance Science, by providing insights into human performance across a broad spectrum of activities and contexts. The intersection of scientific disciplines (e.g., psychology, cognitive science, human movement science, musicology, anthropology, medicine), performance domains (e.g., sport, music, dance, education), and scientific methodologies (e.g., neurophysiological, psychophysical, behavioral) pursues evidence-based knowledge with relevance for scientists and practitioners alike. The disparate nature of these component disciplines and the widespread applications in contexts far beyond academia make Performance Science a radically interdisciplinary area of human knowledge (e.g., Collins et al., 2011; Danielsen et al., 2023; Tod and Eubank, 2020).

## 2 Overview of articles

**Figure 1:**
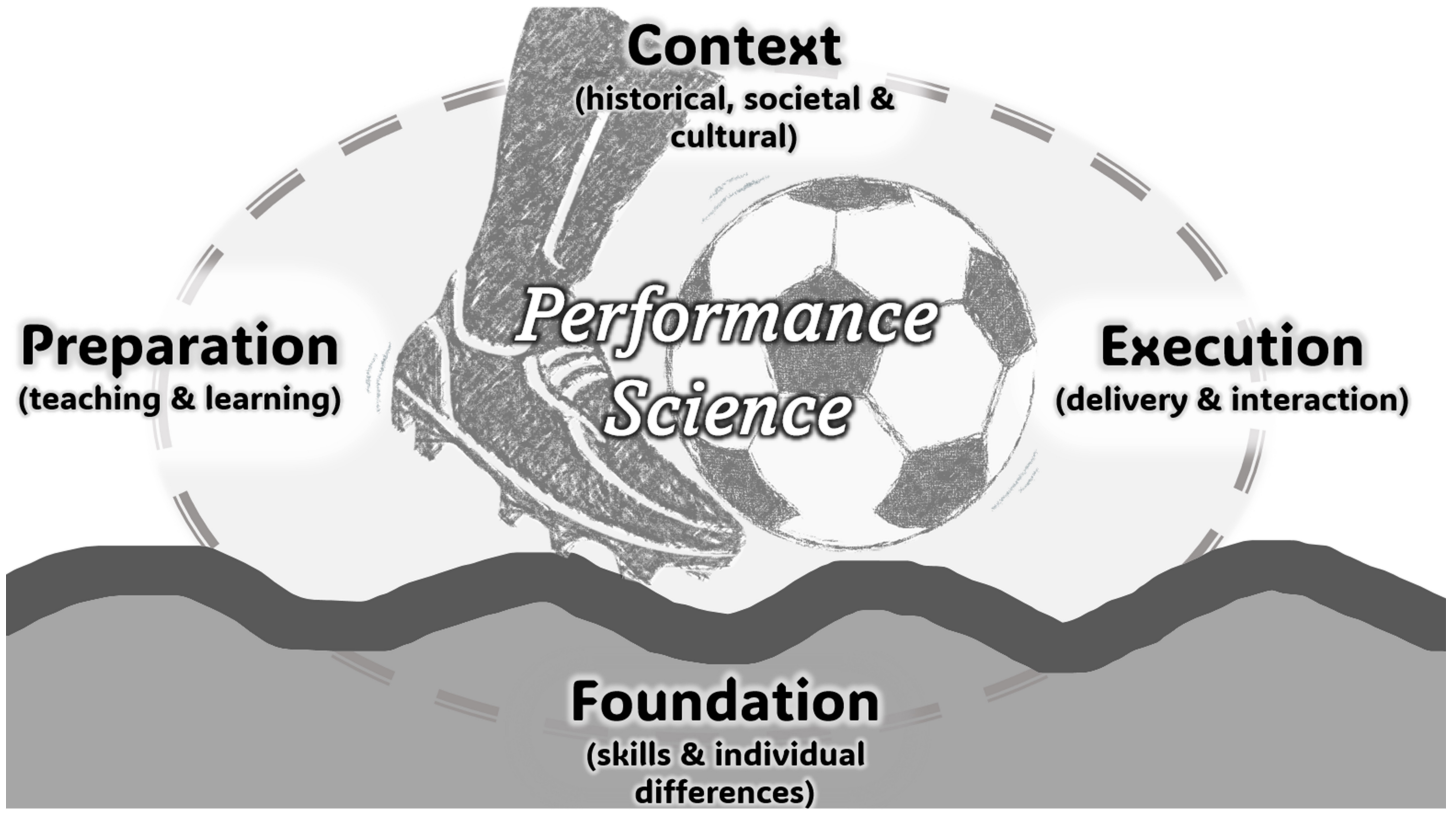
A conceptual framework of Performance Science, indicating four interconnected thematic areas. Taking soccer as an example in the performance domain of sport, for the actual performance execution, performance can be prepared under a learning regime. This occurs within a societal, cultural, and historical context, based on a foundation of an individual skillset.

The featured contributions can be classified into four thematic areas (Figure 1)^1^: Performance foundation (i.e., individual differences and skills); Performance preparation (i.e., teaching and learning); Performance execution (i.e., delivery and interaction with co-performers and audiences); and Performance context (i.e., historical, societal, and cultural contexts within which performance is embedded). We will now introduce these one by one.

### 2.1 Performance foundation

Individuals come to performance situations equipped with their own backgrounds and skills. Contributions in this category focus on timing behavior in rhythmic tasks, acoustics of vocal performance styles, and the neuroscience of academic performance.

By comparing beat alignment abilities of active musicians to those of inactive musicians and non-musicians, Spiech et al. find that active musicians possess significantly stronger beat alignment abilities, with years of formal musical training as the only significant predictor of beat perception.

In a systematic review on Spontaneous Motor Tempo (SMT) in healthy adults, Desbernats et al. highlight that the reference means for SMT are far from universal; rather, a range of SMT values exists, and many factors modulate them. The authors discuss these modulators according to a classification as intrinsic factors pertaining to personal characteristics (e.g., pathology, age, effector, expertise) and extrinsic factors pertaining to environmental characteristics (e.g., physical training, external constraints, observation training, type of task).

Kim investigates the dynamics of intentional sensorimotor desynchronization by asking participants to tap increasingly ahead of a metronome cue. An adaptive-frequency oscillator model captures tapping performance, revealing that desynchronization is governed by the non-linear dynamics of rhythmic coordination.

Bruder and Larrouy-Maestri examine highly trained female classical singers' ability to adjust their vocal production to various styles including their own formal training area (as an opera aria, pop song, or lullaby). Distinct acoustic profiles emerge, implying that singers can indeed produce contrasting performances. In a subsequent perceptual experiment, lay listeners' high accuracy in recognizing the performed singing style confirms singers' proficiency in performing in operatic style, and their versatility when delivering lullaby and pop performances. This performance competence is, however, not linked to singers' formal training levels.

Xu et al. record electroencephalogram (EEG) during resting state, working memory, and general intelligence tasks, in college students with different levels of academic performance. Their results reveal a reduction in alpha-band activity (8–9 Hz) connecting the frontal and occipital lobes in low-performing students, compared with high- and average-performing students, in the general intelligence task. Possible explanations include fatigue, anxiety, and cognitive effort.

### 2.2 Performance preparation

Performance skills are mainly acquired through training. Several articles aim at enhancing our understanding of the mechanisms sustaining efficient teaching, and the importance of learning and practice for optimized skill acquisition.

Combining perspectives from ecological psychology and embodied cognitive science, Miura and Seki demonstrate how physical touch has beneficial effects on motor skills learning in classical ballet. Specifically, touch becomes an effective way of embodying teachers' verbal instructions.

Tuyà Viñas et al. investigate the effects of decision-making on movement variability and passing accuracy in high-level female soccer players, during an elastic band resistance task. Adding decision-making to a forward-backward movement task with ball and elastic resistance increases the movement variability of defenders, but not of attackers. Attackers' passing accuracy, on the other hand, is reduced. Deliberate consideration of decision-making constraints may thus enhance soccer training.

In a study on expert percussionists' choices between left- and right-hand sticks for sight-reading of timpani parts, Bacon et al. demonstrate a prominence of two overall strategies (“dominant-hand lead” and “alternating”) when rhythms are stable, and notation is as expected. However, as excerpts become more musically and/or visually challenging, sticking choices amongst percussionists tend to diversify.

Moen et al. present a study utilizing Bio-Electro-Magnetic-Energy-Regulation (BEMER) therapy to enhance recovery in elite female soccer players on game nights characterized by taxing peaks in physical load. Continuous sleep monitors reveal substantial disturbance in sleep following game nights, which may be alleviated by persistent use of BEMER therapy.

### 2.3 Performance execution

A number of contributions focus on aspects of actual performance delivery. These involve interactions between co-performers and between performers and audiences, with an emphasis on flow and bodily expression.

Zielke et al. investigate the precise musical features that induce or disrupt flow in music performance. They find that the proportion of performance time spent in flow significantly correlates with self-reported flow intensity. This provides a novel intrinsic measure of flow. The authors further analyze music scores and performed melodies, finding that stepwise motion, repeated sequence, and a lack of disjunct motion are common to flow-state entry points, whereas disjunct motion and syncopation are common to flow-state exit points.

Gibbs et al. assess inter-subject correlations in heart rate variability and skin conductance, measured in novice and expert players of Central-Javanese gamelan music. Whereas, expert players exhibit greater physiological synchrony when playing traditional pieces over (stylistically unfamiliar) improvised playing, novice players show the opposite pattern. Furthermore, in traditional playing, these physiological measures relate negatively to a self-report measure of shared flow, whereas the association is positive for improvised playing.

Vukadinović advocates a deeper appreciation of performative aspects of hair and hairstyle as a means of expression in dance. As such, hair can be suppressed (as in the “aesthetics of order” found in the classical ballet bun); used decoratively (as in the ponytails of Broadway and Ballroom dancers); used metaphorically, to express, for example, strength, sensuality, and freedom (as in the long, loose hair of contemporary flamenco dancers); or used instrumentally, to enhance the spectator's perception of liveliness, vibrancy, and stillness.

D'Amario et al. examine the effects of perceived voice-matching with a co-performing singing partner and the complexity of the singing task (unison and canon) on body motion. The authors collect upper-body movement of advanced choristers singing duos along with a recording. Results show that the settings perceived as least together relate to extreme differences between the spectral components of the sound. In addition, compared to canon, singing in unison promotes more periodic movements, more open upper-body posture, and more distance from the music stand.

### 2.4 Performance context

How performance is defined, how it is achieved, and how it is assessed strongly depend on the context in which it manifests. Several studies focus on the historical, societal, and cultural environments that underpin performances.

Hadar and Rabinowitch tackle the social dynamics characterizing different musical genres. They select four genres occupying an extensive continuum of music performance and transmission styles, from strictly orally transmitted and/or notated to fully improvised music. Their new framework examines the social meanings of musical performance styles in terms of structural sparseness, flexible social roles, cultural non-conformity, and creative freedom.

Upham et al. investigate fans' Twitter engagement during live-streamed concerts of the K-pop group BTS, analyzing the content patterns in 1,200 tweets. Postings during these performances appear to be principally directed toward fellow fans and audience members. Individuals choose to share their excitement and check in with other concert attendees, to construct a collective and interactive concert space and create a richer experience.

Based on Ecological Systems Theory, Thuany et al. conceptualize the factors contributing to runners' performance. Their holistic approach allows the identification of environmental determinants which otherwise have been limited to individual characteristics (e.g., in physiology, genetics, and biomechanics).

Finally, Lee et al. compare research interests and trends in soccer-related journal articles published before vs. during the COVID-19 pandemic. A new focus on developing fast-paced, highly efficient training sessions emerged as a response to imposed constraints, along with a switch in focus from head injuries to lower-limb injuries and new concerns about the economic impact of the COVID-19 pandemic.

## 3 Concluding remarks

The present Research Topic illustrates the diversity and creativity of research approaches within the advancing field of Performance Science. The interconnected thematic areas of foundation, preparation, execution, and context depicted in Figure 1 can serve as a conceptual framework for research within different performance domains, be it music, dance, sport, or education. Individual differences and skills, teaching and learning, implementation and interaction dynamics, and contextual characteristics can all be considered essential components in the conceptual architecture of performance. This applies to novices as well as to expert performers. Furthermore, the fact that performance domains are directly related to everyday contexts and experience naturally imbues Performance Science research with high degrees of ecological validity, and applicability in wider contexts and across individuals.

To investigate such complexity, future research will benefit from transdisciplinary dialogue across scientific domains and thematic areas. The current Research Topic represents merely a glance into the possible future of Performance Science (cf. Filho and Basevitch, 2021). This glance is far from exhaustive; for example, research on performance in the healthcare sector, the workplace or sport coaching was not fully represented. Yet, we envision that theoretical frameworks, methodologies, and findings from the Performance Science domains covered in this Research Topic may also prove useful within these knowledge areas (cf. Aoyagi et al., 2012; Sandars et al., 2022; Schiavio et al., 2019). Finally, although the contributions to this Research Topic focus mostly on achieving specific goals and performance outcomes (e.g., delivering a concert, executing movement sequences, or rehearsing routines), performance scientists should not neglect the health and wellbeing of performing individuals, which may indeed be compromised in multiple ways during pursuits of human performance (cf. WHO, 2024; Williamon and Antonini Philippe, 2020).

## Footnotes

^1^Figure 1 uses visual materials from vectorportal.com (boot, CC-BY 4.0) and rawpixel.com (ball, CC0 1.0) that have been modified for the present use.
